# Contactless continuous heart rate monitoring system using ballistocardiography

**DOI:** 10.1371/journal.pone.0272072

**Published:** 2022-07-29

**Authors:** Brian Sumali, Yasue Mitsukura, Toshihiko Nishimura

**Affiliations:** 1 Keio Global Research Institute, Faculty of Science and Technology, Keio University, Yokohama, Kanagawa, Japan; 2 Department of System Design Engineering, Faculty of Science and Technology, Keio University, Yokohama, Kanagawa, Japan; 3 Department of Anesthesia, Stanford University School of Medicine, Stanford, California, United States of America; Universita degli Studi di Pisa, ITALY

## Abstract

Cardiovascular disease is the number one cause of death in the world and is a serious problem. In the case of cardiopulmonary arrest due to myocardial infarction, the survival rate is as low as 13.3% one month after resuscitation, which birthed the need for continuous heart monitoring. In this study, we develop a Ballistocardiogram (BCG) measurement system using a load cell installed on a chair and a heart rate estimation algorithm that is robust to waveform changes, with the aim of constructing a non-contact heart rate acquisition system. The proposed system was evaluated by utilizing data obtained from 13 healthy subjects and 1 subject with abnormal ECG who were simultaneously measured with ECG. The output of the BCG system was confirmed to change with the same period as the ECG data obtained as the correct answer, and the synchronization of the R-peak positions was confirmed for all cases. As a result of comparing the heart rate intervals estimated from BCG and those obtained from ECG, it was confirmed that the same heart rate variability (HRV) features could be obtained even for abnormal ECG subject.

## 1. Introduction

In recent years, heart disease has become the number one cause of death in the world and is a serious problem [1]. In addition, even if a patient is resuscitated after cardiopulmonary arrest due to cardiac disease, the survival rate after one month is as low as 13.3% [2]. Early detection of heart disease can be actualized by daily monitoring as persons with heart disease have abnormal heartbeats.

The heart rate is affected by the autonomic nervous system, which is constantly changing due to both physical factors such as exercise and diet, and mental factors such as stress. The autonomic nervous system is controlled by two contradictory nerves, the sympathetic and parasympathetic, and fluctuates depending on the degree of equilibrium between these nerves. In general, the heart rate decreases with age, and this is thought to be due to the sympathetic dominance of the balance between the two nerves, which is associated with heart disease. In fact, 83% of all cardiac deaths occur in people aged 65 years or older, and monitoring heart rate information reflecting the autonomic nervous system was shown to be effective in preventing cardiac diseases [3].

Although the gold standard for obtaining electrical signals from the heart in clinical settings is electrocardiography (ECG), the ECG devices are relatively expensive and inaccessible to the public. In recent years, wearable devices have been attracting attention as an alternative method to acquire heart rate information [4–6]. The popularity might be related to its effectiveness for obtaining heart rate information for the whole day and the convenience of attaching a small wearable device to the body. Some of the commercial devices, however, are unsuitable for clinical diagnosis as they do not have the clinical validation study.

One of the alternatives is ballistocardiogram (BCG). The BCG is a signal obtained by using the reaction of the blood pushed out when the heart beats, and it is known that there is a relationship between the heart signals obtained by ECG and the signals obtained by BCG [7, 8]. Other than heart rate, BCG can also be used to monitor blood volume, respiration, and other characteristics [9–11]. BCGs, however, are not without disadvantages. As the measurement device is not directly attached to the body, artifacts caused by body movement and posture can change the recorded signal, reducing its quality.

**Fig 1:**
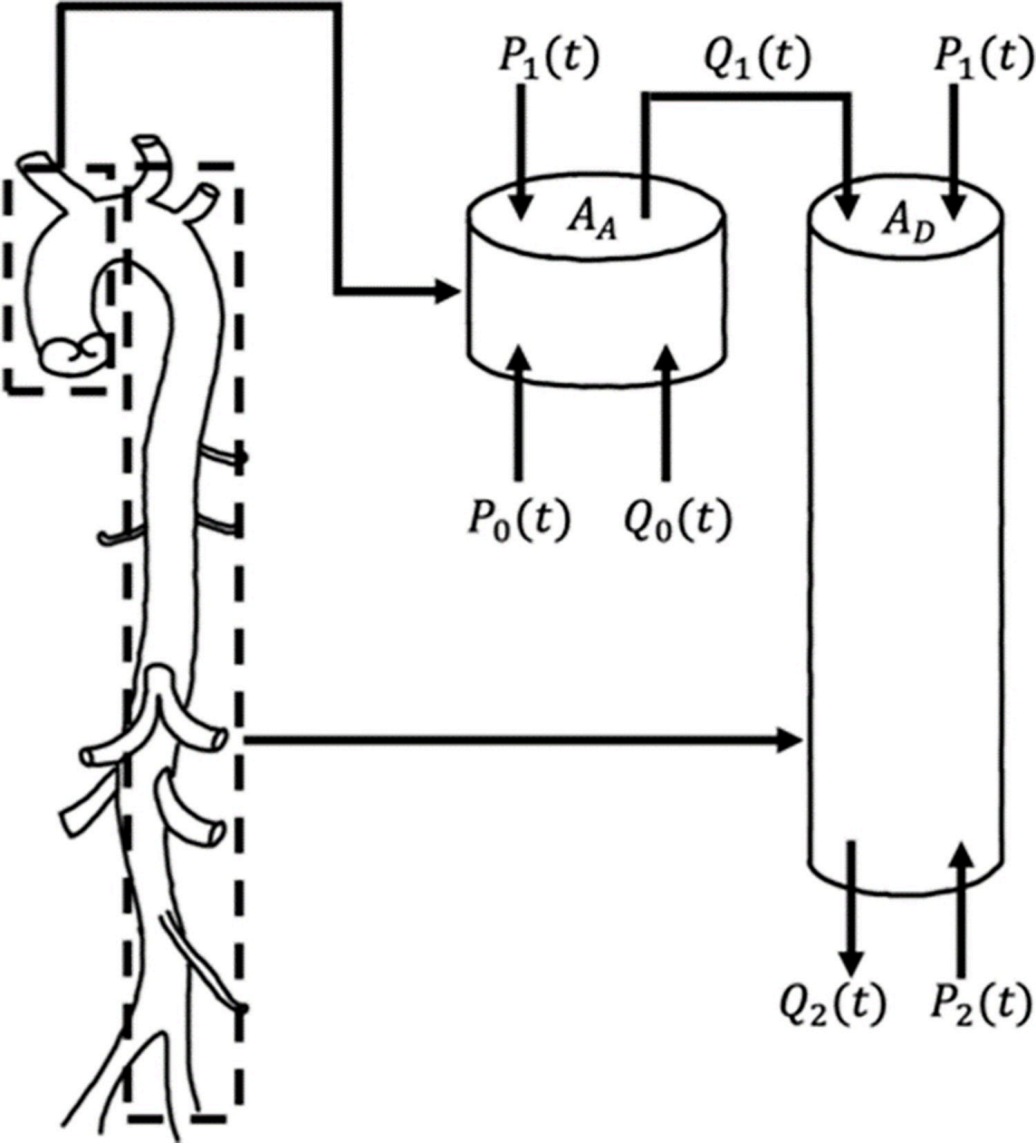
Model of heartbeat mechanism and changes in force [7].

The BCG acquires the heartbeat as a vibration using its reaction. The vibration is acquired indirectly by a load sensor attached to a chair or bed. The BCG reflects the reaction of the center of gravity change associated with the contraction and relaxation of the heart and the pumping of blood, as shown in Fig 1. Among these, the effect of the change in the center of gravity due to blood pumping is particularly significant. By modeling the force change in the vertical (head-foot) direction of the body, with the force acting in the head direction as positive and the force acting in the foot direction as negative, the following equation can be obtained.


$$F_{BCG}(t)=A_{D}[P_{1}(t)-P_{2}(t)]-A_{A}[P_{0}(t)-P_{1}(t)]$$

Here, *A*_*A*_ and *A*_*D*_ represent the mean cross-sectional area of the ascending and descending aorta, respectively, *P*_0_(*t*) represents the blood pressure at the aortic inlet, *P*_1_(*t*) represents the blood pressure at the boundary between the ascending and descending aorta, and *P*_2_(*t*) represents the blood pressure at the outlet of the descending aorta.

Cardiac activity is transmitted in the order of *P*_0_(*t*), *P*_1_(*t*), and 2(*t*). Therefore, when the heart contracts, *P*_0_(*t*) increases first, and then *F*_*BCG*_(*t*) decreases. This causes the I wave to appear. After that, *P*_1_(*t*) increases, *F*_*BCG*_(*t*) increases with the increase of *F*_*BCG*_(*t*), and J wave appears. Finally, *F*_*BCG*_(*t*) decreases with the increase of *P*_2_(*t*), and K wave appears.

An even specialized model of heartbeat mechanism is described in [12]. The paper describes the blood flow through the complete circulatory system. In this study, the most significant contributor to the BCG signal are the blood flow in the abdominal aorta.

BCG has been actively researched and there is a growing trend to obtain heart rate information for clinical purposes. Although obtaining a ECG-like signal from BCG is still a challenge, studies have successfully extracted heart-rate variability (HRV) features from BCG signals [8]. Traditionally, HRV features are extracted from R-R interval (RRI) information, obtained from ECG signals and such features have been shown to be effective for understanding both physiological and psychological events along with diagnosing cardiac events.

Many types of BCG sensors have been proposed. Heise et al [13, 14] constructed a BCG measurement system with a hydraulic sensor under a mattress while Watanabe et al [15] pneumatic sensor and obtained the user’s heart rate, respiration rate, snoring, and body movement. Fiber optics [16, 17], Electromechanical Films (EMFI) [18], and strain gages have also been employed [19, 20] as BCG sensors.

A strain gage has a structure in which resistance wires are reciprocated, and when the material to which it is attached expands or contracts, the resistance wires also expand or contract, causing the resistance value to change. Using this mechanism, strain gage sensors are installed in the legs of chairs, beds, and scales to obtain biological signals from the changes in load applied to the sensor. This flexible installation points allows for continuous measurement with minimal obstruction or changes from daily lives.

In this study, the development of a continuous heart-rate measurement system with minimal quality-of-life obstruction while being robust to environmental noise. As such, strain-gage based sensors were selected as the main sensor in this study and the placement location is set to be under the chair legs. The main contributions of this paper are (1) better R-peak detection from BCG signal and (2) a proposal of improved HRV calculation method from BCG signal.

## 2. Materials and methods

### 2.1. Data acquisition

This study was approved by the Bioethics Committee of Faculty of Science and Technology, Keio University with approval ID 31–78. Recruitment was performed in Japan by the authors to their acquaintances. Thirteen healthy subjects and one abnormal ECG patient voluntarily participated in this study. All healthy subjects were Asian in their 20s (average age 22.9±0.7 year), while the patient is their 40s. Everyone provided written informed consent. The experiment was conducted on a typical room in Keio University Faculty of Science and Technology, Yagami Campus.

During the recording session, the participant is asked to sit on a four-legged round chair. The load-cell sensor is fitted under each leg and then asked to stay calm and relaxed for ten minutes. ECG is also recorded as a gold-standard measurement.

### 2.2. Materials

**Fig 2:**
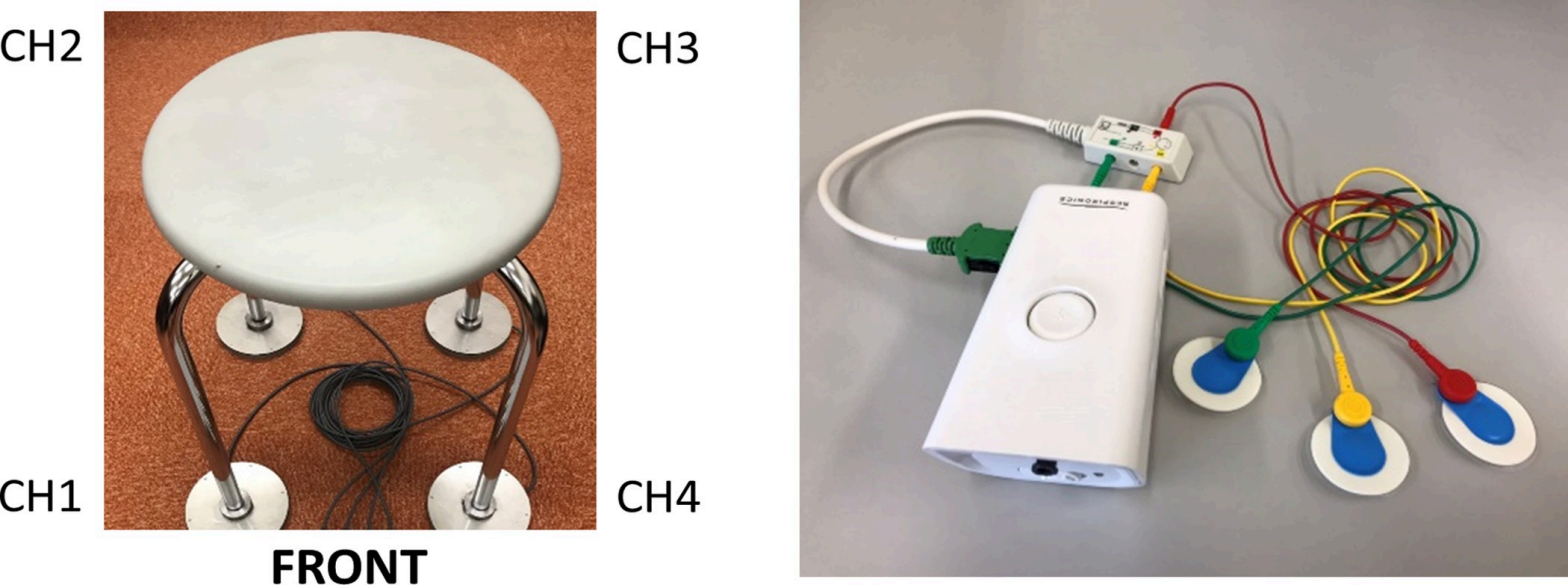
The recording devices; (a) Chair fitted with load-cell sensors; (b) Alice PDx.

BCG sensors: Four load-cell sensors were attached to the four-legged round chair, as shown in Fig 2(A). The load-cell sensors were custom-made, and the data logger used in this study was CONTEC AIO-160802AY-USB and the sampling rate was 200Hz. A load-cell sensor is an array of four strain gages, electrical sensor which convert “strain” (force, weight, pressure, etc.) applied to it into electrical voltages. These sensors were custom made and they function as BCG by detecting miniscule vibrations caused by the beating heart. Differing from seat-type BCG sensors, by employing four load-cell sensors at the chair legs, the system offers stability during cases when the mass isn’t distributed equally, especially by applying the preprocessing steps described in the following sections.

**Fig 3:**
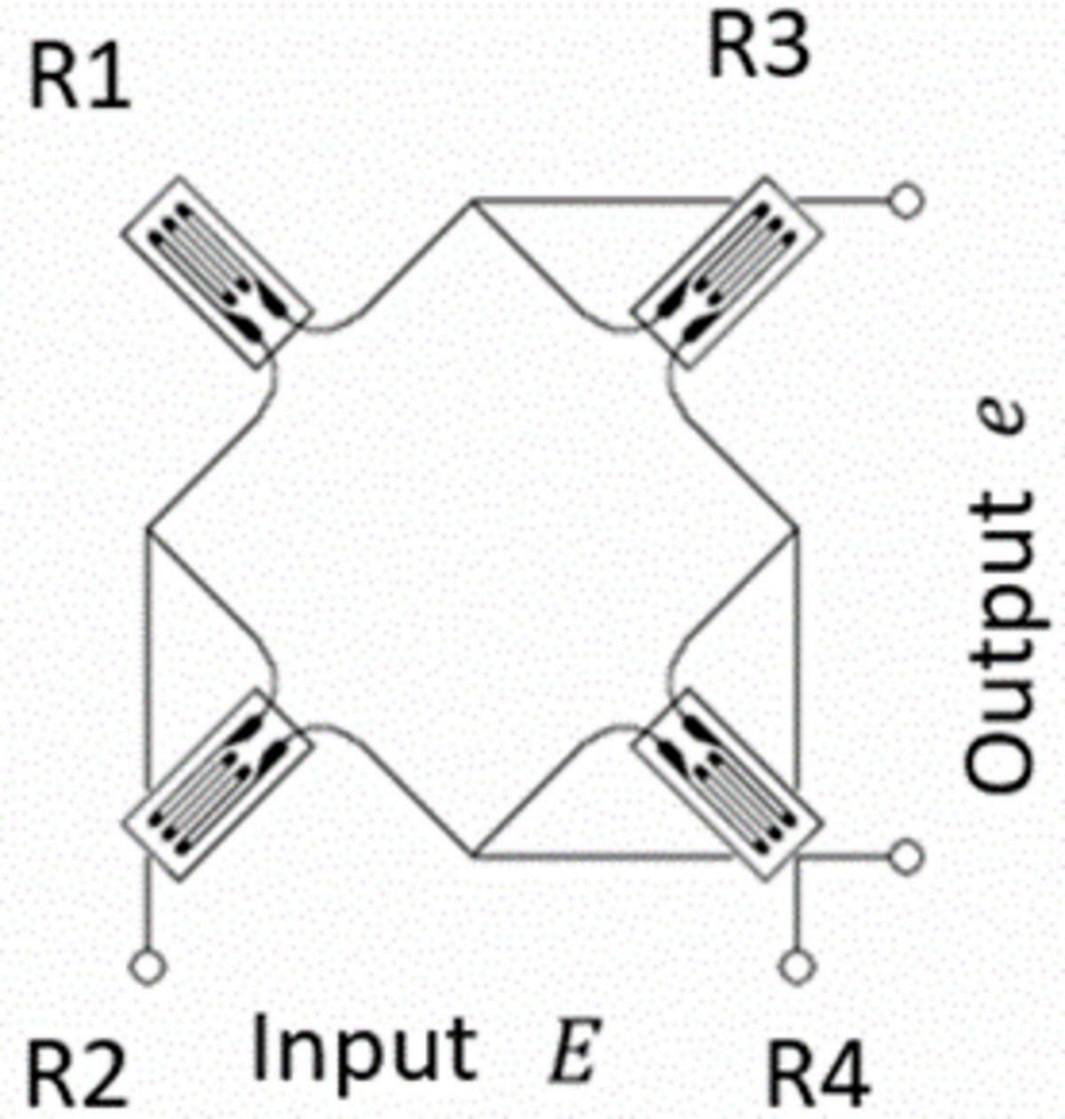
The wheatstone bridge arrangement of load-cell sensor.

Each load cell sensor employed 350Ω resistors with the wheatstone bridge arrangement, shown in Fig 3 The Wheatstone Bridge arrangement of load-cell sensor. The outputs were computed as follows:

$$e=\frac{R_{1}R_{3}-R_{2}R_{4}}{\left(R_{1}+R_{2})(R_{3}+R_{4}\right)}E$$

Here, e, E, R1, R2, R3, and R4 indicate Output, Input, and the resistance values, respectively.

ECG sensors: Ambu® BlueSensor ECG Electrodes with Philips’ Alice PDx, as in Fig 2(B). The sampling rate was set to 200Hz.

### 2.3. Analysis

**Fig 4:**
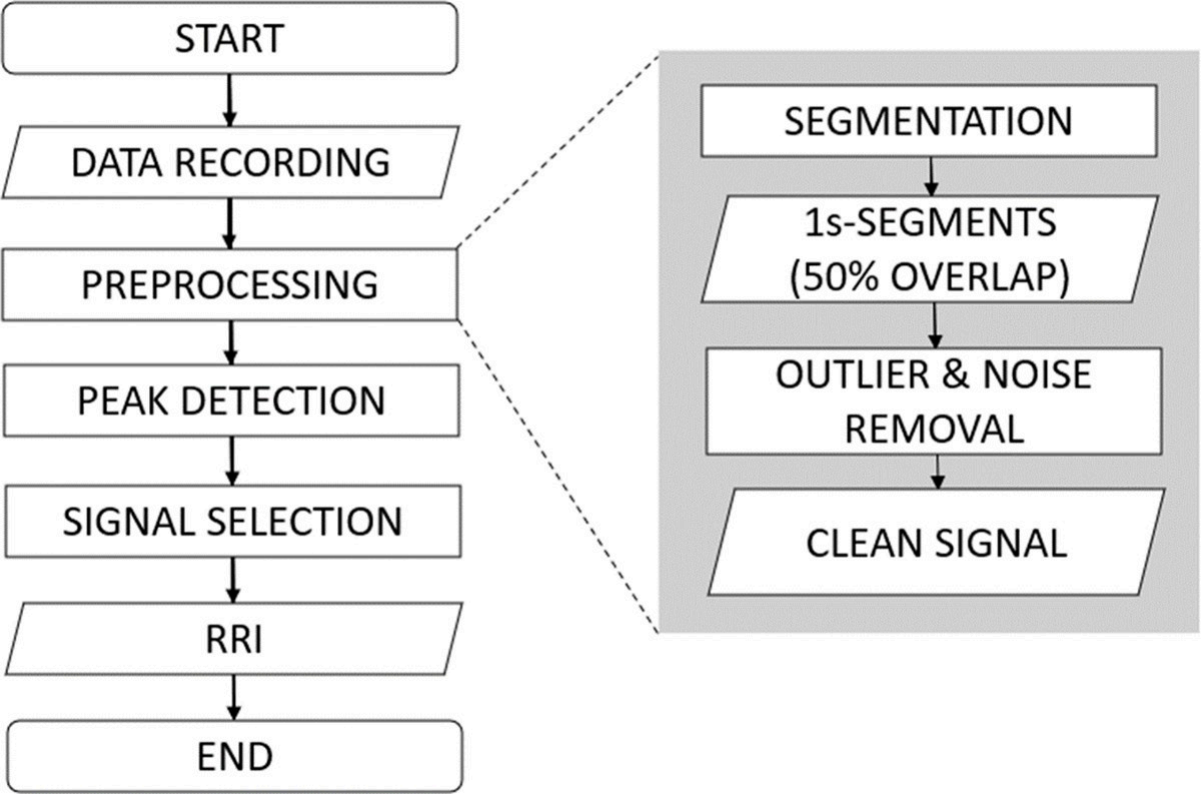
Analysis flowchart. Validation is based on the output RRI.

The flowchart of BCG signal analysis and its validation is provided in Fig 4. All signals from four channels are processed, until one is finally selected in the “signal selection”.

#### 2.3.1.Preprocessing

*Data segmentation*. The aim of this preprocessing step is to remove outliers and attenuate noise. To simplify the outlier removal step, the data is first segmented into multiple short-segments by splitting them by 1-second window with 50% overlapping segments.

*Amplitude thresholding*. Segments with 85mV or higher amplitude were defined as segments contaminated with heavy noise and segments with 35mV or lower were defined as segments without proper body-to-chair contact. These segments were marked and then removed from the analysis and the total amount of remaining segments utilized for analysis was 58699 segments.

*Filtering*. Bandpass filter of 1Hz to 9Hz were applied to remove high frequency noise and respiratory components.

#### 2.3.2. Peak detection

**Fig 5:**
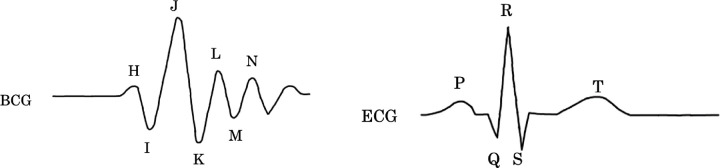
Sample BCG and ECG signals showing the correspondence between R-peaks and J-peak.

Accurate prediction of R-peak occurrences is very important for determining the HRV features. We showed the illustration of the BCG and ECG signals in Fig 5 Sample BCG and ECG signals, for the purpose of showing the correspondence between R-peaks (ECG) and J-peaks (BCG).

From the segments obtained in the previous steps, the local extrema were calculated to extract the candidates of R-peaks. Machine learning with Convolutional Neural Network were performed as the next step. Three seconds worth of BCG signal with the extrema points as the center are utilized as the input. The output for the CNN is the location of R peak from the similar segment from ECG.

After sufficient training (i.e. the stopping criteria of error < 0.0001 and absolute margin < 0.0001 were reached), the CNN was able to accurately predict the timing of the R-peaks from a given BCG segment. The timings of the estimated R-peaks (from J-peaks) were obtained from BCG segments using the trained CNN model and the HRVs were computed from the obtained R-peaks.

#### 2.3.3. Signal selection

It is known that the RRI varies from 0.6s to 1.0s in normal conditions, as adult heartbeat ranges from 60–100 beats per minute. Therefore, the deviations from the normal conditions can be detrimental for the signal quality. These deviations are primarily caused by the unequal weight distribution caused by the sitting position of the user. Thus, a simple thresholding is performed to measure the signal quality. Segments which satisfy the following requirements are marked as bad segments:

- Minimum value: 0.5s
- Maximum value: 1.1s

The channel with the least number of bad segments is selected for representing the subject’s HRV.

#### 2.3.4. Validation

As a benchmark, the conventional algorithm [20] is selected as it was the closest algorithm that resembles our proposed method. In principle, the inputs and pre-processing methods are similar for both the proposed and the conventional method, differing mainly in parameter choice and the bandpass range. The main difference between conventional method and the proposed method lies in the peak detection algorithm, where the conventional method used peak detection algorithm while we opted to use CNNs to predict the J-peaks. The structure of the CNN we utilized in this study is described in the following pseudocode supplied in the supplementary materials.

Effectiveness of the proposed method is validated twofold: first, by comparing the error between the predicted R-peaks and the actual R-peaks from ECG and second, by comparing the error between the predicted HRVs and the actual HRVs from ECG. For the R-peak comparison, accuracy and recall of the CNN were also measured with leave-one subject out cross-validation. The data employed for both training and validation were from the thirteen healthy volunteers. The data obtained from patient with abnormal ECG were used solely as a separate validation session.

Per each 1-window epoch, the predicted R-peaks timings and true R-peak timings were compared. If the timing is correct within 0.1s, the epoch is considered as correctly predicted. Otherwise, it is considered as false. Then, the accuracy and recall were computed with the following equations:

$$Acc=\frac{TP+TN}{TP+TN+FP+FN}$$

$$Recall=\frac{TP}{TP+FN}$$

where Acc, Recall, TP, TN, FP, and FN indicates accuracy, recall, true positives, true negatives, false positives, and false negatives, respectively. Finally, the performance was also validated by applying the proposed algorithm to post-exercise data and the errors were computed.

## 3. Results

**Fig 6:**
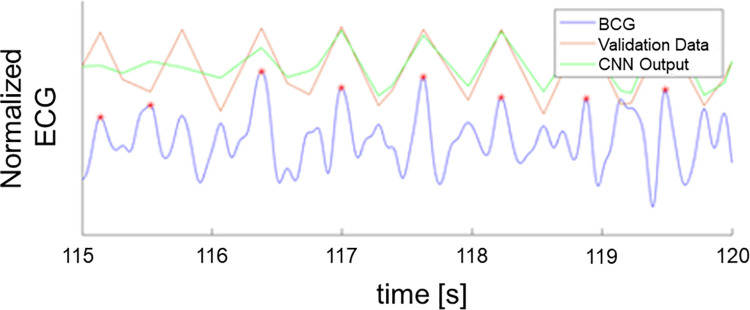
R-peaks estimation using CNN vs R-peaks from ECG signal.

**Fig 7:**
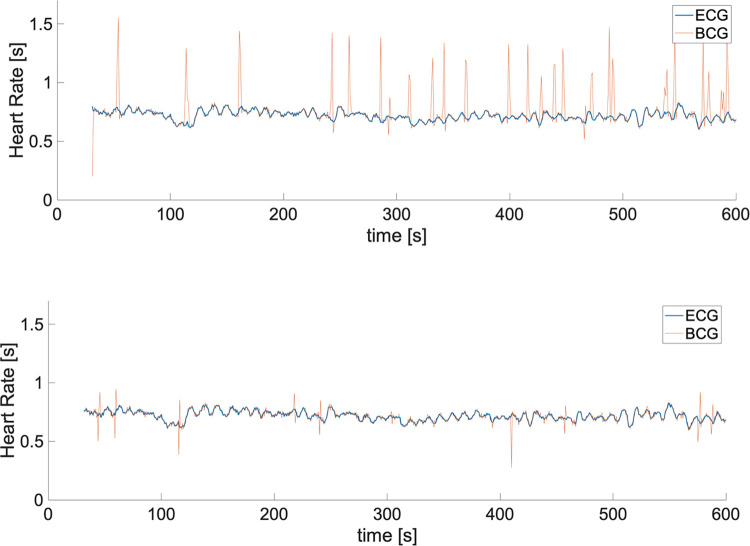
Comparison of HRV obtained from conventional algorithm and proposed method; (a) HRV obtained from conventional algorithm [21]; (b) HRV obtained from proposed method.

**Fig 8:**
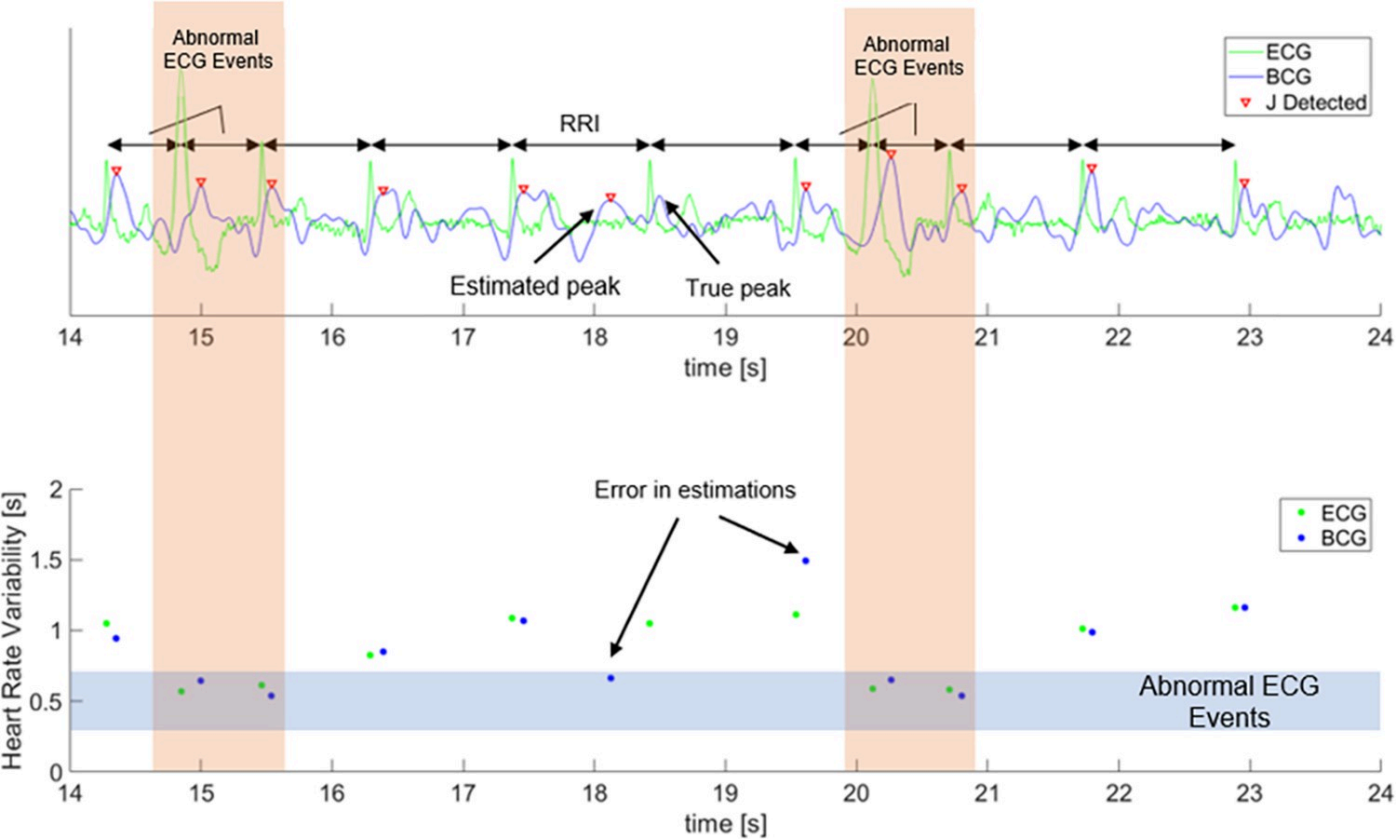
Accurate detection of R peaks from subject with abnormal ECG waves. The occurrences of irregular heartbeats are shown with orange background.

**Fig 9:**
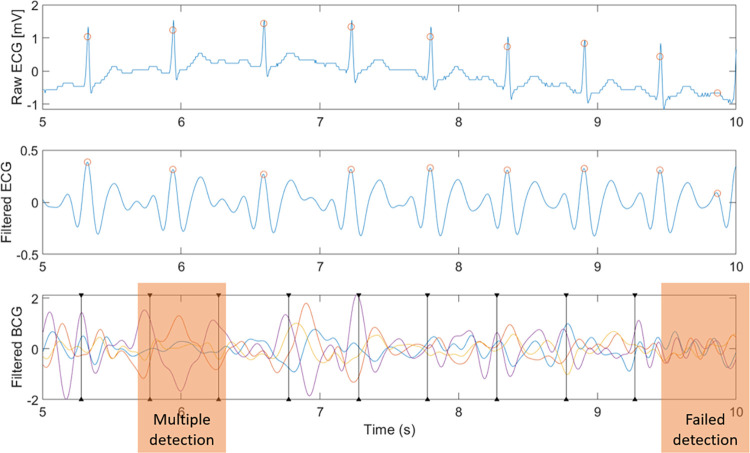
An example of detection of R-peaks directly after the exercise period; The orange boxes note indicate failures in peak detection.

The result from the healthy subjects is shown in Fig 6 which show the comparison of extracted R-peaks from normalized ECG and BCG signals. The proposed method-predicted R-peaks with accuracy of 95.9% and recall 95.3%. The error rate of the proposed method is 2.07±0.27 bpm, which is better than the conventional method [20] of 10.12±4.69 bpm. The sample of HRVs extracted from conventional method and proposed method is also available in Fig 7. As an additional validation, both the proposed method and the conventional method [20] were tested against abnormal ECG patient. The example of the proposed method accurately predicting HRVs in abnormal ECG events is shown in Fig 8. Mean error of 0.85 ± 11.98 bpm was obtained when the errors were computed from post-exercise data, as in Fig 9.

## 4. Discussion

As shown in the results, the proposed method was more effective at extracting HRV features compared to the conventional method [20]. Additionally, although it was trained and adjusted for healthy subjects, the resulting model is still able to accurately perform for patients with abnormal ECG.

HRV is known to be affected by diet, exercise, breathing, stress, sleep, etc. and it is also known to be related with cardiovascular diseases, especially ischemic heart diseases. Ischemic heart disease is a disease in which the coronary arteries become blocked due to clogged blood vessels or other causes, cutting off the supply of oxygen and nutrients to the heart muscle. While normal blood vessels have a structure that repeatedly branches and joins, coronary arteries rarely have joining points, so blockage upstream can lead to severe ischemic heart disease. The sympathetic and parasympathetic nervous systems, which make up the autonomic nervous system, also act antagonistically in the heart. It is said that should the parasympathetic nervous system be disturbed; the electrical stability is disrupted, and arrhythmia occurs as a result. When the parasympathetic nervous system is affected, the heart rate variability decreases and the resting heart rate increases, making it possible to detect signs of ischemic heart disease by observing the heart rate variability.

The results confirm that the proposed algorithm performed effectively even for users with irregular heartbeat. Furthermore, the accuracy and precision of the estimated HRV feature might suggest the possibility of implementation in clinical scene, although further experiment with more study participants is needed to solidify this claim. Nevertheless, the performance of the proposed algorithm is severely worse when using the post-exercise data compared to the resting state, with very high error variances.

This study is not without limitations. Since the number of patients with abnormal ECG is only one and the patient was not in relative cardiac danger, the effectiveness of the proposed system in real world situation remains inconclusive. Additionally, although it was tested with leave-one subject cross-validation, the sample size of participants is small, and the age variance is also small. Furthermore, as BCG is a physical sensor, physical properties of the participants such as their height, weight, BMI, etc. should affect the performance of the sensors and are an important factor to consider. These limitations must be seriously considered especially when applied to patients with irregular heartbeats or cardiac infarctions and elderly users. The proposed system shows the possibility of non-contact heart acquisition system for healthy adults and one arrythmia adult. It is by no means perfect and further experiment and testing is needed before its application for clinical settings.

## 5. Conclusion and future work

### 5.1. Conclusion

The objective of this study is to propose an improvement for HRV estimation based on BCG signal inputs. During the experiment, the participants were asked to sit in relaxed state for ten minutes. As a result, the proposed method overperforms the conventional method in both accuracy and recall percentages and mean error. The proposed system was also tested in a single abnormal ECG subject and performs unexpectedly well.

### 5.2. Future work

Suggestions for future work is as follows: (1) larger sample size with varying age distribution, to validate the effectiveness of this study for more generalized sample size. (2) larger sample size with cardiovascular diseases is needed, to confirm the effectiveness of the proposed system for non-healthy subjects. Next (3) the utilization of signal fusion technologies instead of signal selection might improve the overall performance of the system. Finally (4) utilization of machine learning models specialized for peak detection might also improve the performance; as in this study, CNN improves the system’s effectiveness compared to the conventional studies using conventional peak detection algorithm.
